# Why study moonlighting proteins?

**DOI:** 10.3389/fgene.2015.00211

**Published:** 2015-06-19

**Authors:** Constance J. Jeffery

**Affiliations:** Department of Biological Sciences, University of Illinois at ChicagoChicago, IL, USA

**Keywords:** moonlighting proteins, multifunctional, protein evolution, protein structure and function, enzyme function

## What are moonlighting proteins?

**Figure 1:**
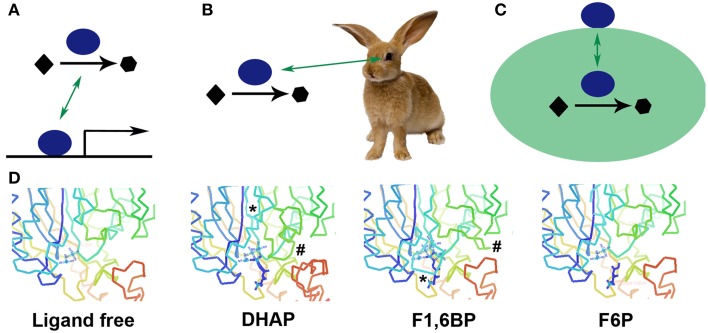
**Cartoons illustrating how a protein can moonlight**. A protein can catalyze an enzymatic reaction and also **(A)** bind to DNA and serve as a transcription factor, **(B)** have a noncatalytic role in the lens of the eye (crystallins), or **(C)** be placed on the surface of a cell to function as a receptor. Another type of moonlighting is illustrated in **(D)** where an active site undergoes significant remodeling, or movement of loops (marked by ^*^ and #) to bring in different catalytic amino acids to perform different two different catalytic functions. **(D)** made Using Coot (Emsley et al., 2010) and PDB IDs 3T2B, 3T2C, 3T2D, and 3T2E.

Moonlighting proteins comprise a class of multifunctional proteins in which a single protein performs multiple physiologically relevant biochemical or biophysical functions that are not due to gene fusions, multiple RNA splice variants, or pleiotropic effects (Jeffery, 1999). Classic examples include soluble enzymes that also bind to DNA or RNA to regulate translation or transcription (Figure 1A) (reviewed in Commichau and Stulke, 2008) or have a second function as structural proteins in the lens of the eye (crystallins) (Figure 1B) (Wistow and Piatigorsky, 1987; Piatigorsky and Wistow, 1989). Other typical examples are cytosolic enzymes that moonlight as cytokines, chaperones, cytoskeletal components, DNA compactors, adhesins or scaffolds, as well as a chloride transporter that regulates the function of another transmembrane channel, ribosomal proteins that double as translation factors, and a DNA binding protein that becomes a component of the extracellular matrix (for reviews see Nobeli et al., 1996; Jeffery, 2003a, b, 2009, submitted; Piatigorsky, 2007; Gancedo and Flores, 2008; Huberts and van der Klei, 2010; Henderson and Martin, 2011, 2013; Guo and Schimmel, 2013). Moonlighting proteins are found in mammals, yeast, worms, bacteria, plants, viruses, archea and many other organisms. The online MoonProt Database, which includes information about those moonlighting proteins for which biochemical or biophysical evidence supports the presence of at least two biochemical functions in one polypeptide chain, includes over 270 proteins (Mani et al., 2015). It is likely that many other proteins also have additional functions that have not yet been found. In this opinion piece, I argue that there are currently many reasons for studying moonlighting proteins.

## Many moonlighting proteins are involved in disease

A growing number of moonlighting proteins have been found to play key roles in disease, but there is space to mention only a few examples here. Phosphoglucose isomerase, an enzyme in glycolysis, is also a cytokine (autocrine motility factor) that plays a role in breast cancer metastasis (Watanabe et al., 1991). Alcohol acetaldehyde dehydrogenase/Listeria adhesion protein (LAP) enables *Listeria monocytogenes* to bind to intestinal epithelial cells and aids in infection (Jagadeesan et al., 2010). Enolase (Knaust et al., 2007; Agarwal et al., 2008; Castaldo et al., 2009), phosphoglycerate kinase (Boone et al., 2011; Fulde et al., 2013), and glyceraldehyde 3-phosphate dehydrogenase (Pancholi and Fischetti, 1992; Seifert et al., 2003; Bergmann et al., 2004; Jobin et al., 2004; Barbosa et al., 2006; Hurmalainen et al., 2007; Matta et al., 2010) are cytosolic enzymes that have a second role in pathogenic bacteria as a cell surface receptor for collagen, fibronectin, or plasminogen (Figure 1C). Adhesion of the pathogen to the host by binding to collagen and fibronectin, components of the host extracellular matrix, aid in colonization of the host. Binding to plasminogen enables its conversion to the active protease plasmin, which is used to degrade host proteins and aid in tissue invasion. In general, these cytoplasmic/cell surface moonlighting proteins can be important in infection, virulence, or immune responses, and some can be potential vaccination targets. Two papers in this Research Topic discuss additional examples of moonlighting proteins involved in disease and how they can be targets for the development of novel therapeutics (Rasch et al., 2015; Henderson and Kaiser, in press).

## Identifying novel biochemical pathways

The growing number of intracellular enzymes or chaperones that are being found to moonlight on the cell surface raises several questions. How do intracellular/cell surface proteins get secreted? How do they become attached to the cell surface? In addition to the well-known Sec pathway, there are several non-canonical secretion pathways, but a secretion method in which a large portion of each protein remains inside the cell while some of it is partitioned to the cell surface has not been identified. This may involve a novel variation of a known secretion pathway or an as yet unknown secretion pathway. In addition, these moonlighting proteins do not contain known signals for attaching to the cell surface. Again, this could involve a new version of a known mechanism or an as yet unknown mechanism for cell surface attachment. With the increasing problem of antibiotic resistance, finding a method to inhibit the targeting of these proteins to the pathogen surface might lead to an alternative method to decrease the ability of bacteria to bind to and degrade host tissues. Understanding more about these cellular processes, whether a new variation of a known process or a completely new process, could provide new target(s) for developing therapeutics to treat infections. Similarly, the study of other moonlighting proteins could lead to the identification of additional previously unknown cellular processes.

## Importance for systems biology

Some moonlighting proteins serve as a connection between multiple biochemical pathways or a switch between pathways, and help the cell to respond to changes in its environment. Many biosynthetic enzymes moonlight as regulators of transcription or translation and serve as a feedback mechanism to regulate the amount of enzyme synthesized in a biochemical pathway in response to changes in the cellular concentration of a product of the pathway. For example, thymidylate synthase is also an RNA binding protein (Chu et al., 1991), and *E. coli* putA is also a DNA binding transcriptional repressor (Wood, 1981; Ostrovsky de Spicer et al., 1991; Ostrovsky de Spicer and Maloy, 1993). Other moonlighting proteins provide a mechanism to switch between biochemical pathways in response to changing cellular conditions. Aconitase/IRE binding protein helps the cell respond to changes in cellular iron concentration (Kennedy et al., 1992; Philpott et al., 1994; Chen et al., 2005; Banerjee et al., 2007). Studies of the structures and functions of moonlighting proteins like these can help elucidate how proteins switch activities in response to changes within or surrounding the cell, whether it's due to changes in pH, substrate availability, cellular concentrations of metal ions, or other factors.

## Novel mechanisms of protein function

Studies of the structures and functions of some moonlighting proteins have already added to our knowledge of the diverse mechanisms by which a protein can perform multiple functions and how protein structure can change in response to changes in its environment (examples in Jeffery, 2004a,b). The results of two recent studies illustrate the sometimes drastic conformational changes that proteins can undergo and add to our understanding of protein structure and function in general.

Two thermophilic fructose-1,6-bisphosphate aldolase/phosphatase enzymes use a single active site pocket to catalyze two reactions in gluconeogenesis, an aldol condensation of dihydroxyacetone phosphate and glyceraldehyde 3-phosphate and dephosphorylation of fructose 1,6-bisphosphate (Du et al., 2011; Fushinobu et al., 2011). Three loops move into and out of the active site bringing in a different set of catalytic amino acids, and altering the binding of several active site metal ions, in order to bind the second substrate and perform the second catalysis (Figure 1D).

RfaH is a transcription factor that interacts with RNA polymerase (RNAP) to reduce pausing and increase processivity, and it is also a translation factor. In order to perform the second task, its C-terminal domain (CTD) undergoes complete refolding (Schweimer et al., 2012). In an all-alpha conformation, the CTD masks the RNAP binding surface on the N-terminal domain (NTD) until the NTD binds specific operons in DNA. Then the CTD is released from the NTD and surface of the NTD can bind to RNAP. The CTD then refolds into an all-beta conformation and recruits ribosomes by binding to the ribosomal S10 protein, to potentiate translation of RfaH-controlled operons, which contain weak ribosome binding sites.

In addition, an increasing number of intrinsically disordered proteins (IDPs) and proteins with intrinsically disordered domains have been found to be moonlighting proteins. The functions of intrinsically disordered proteins often involve interacting with another protein, and the disordered binding region undergoes induced folding upon binding to the protein partner. Some intrinsically ordered domains have different functions by using alternate conformations to interact with multiple protein partners (Tompa et al., 2005). In some cases, these different binding modes and partners result in opposite effects on a pathway, for example by activating one binding partner but inhibiting another.

## Predicting protein functions

Hundreds of thousands of protein sequences are available due largely to the efforts of the genome projects. During annotation of sequence databases, the functions of most new proteins are predicted through amino acid sequence homology to proteins of known function. The ability of proteins to moonlight complicates this procedure.

First, if a protein is homologous to a moonlighting protein, the protein might have both, one, or none of the functions of the moonlighting protein. For example, two versions of aconitase are found in mammalian cells, in the cytoplasm and the mitochondria. Both aconitases catalyze the transformation of citrate to isocitrate in the citric acid cycle, but only the cytoplasmic protein is also an mRNA binding protein. The mitochondrial protein has a different second function in mitochondrial DNA maintenance (Kennedy et al., 1992; Philpott et al., 1994; Chen et al., 2005; Banerjee et al., 2007). Another moonlighting protein, delta 2 crystallin in the duck eye lens is the same protein as the ubiquitous urea cycle enzyme arginosuccinate lyase. The duck delta 1 crystallin, which has 89% amino acid sequence identity to the delta 2 crystallin, does not have catalytic activity (Piatigorsky et al., 1988; Barbosa et al., 1991; Chiou et al., 1991; Graham et al., 1996; Piatigorsky and Horwitz, 1996). Therefore, sequence homologs of moonlighting proteins might not be moonlighting proteins, so sequence homology alone might not be sufficient for correct prediction of protein function.

The second complication is that it is currently unclear how to identify all the functions of a moonlighting protein from its sequence or structure. Many function prediction algorithms aim to identify a single function for a protein. Three papers in this Research Topic and another recent report have used lists of known moonlighting proteins to test current programs and develop novel ways of predicting multiple functions (Khan et al., 2014; Khan and Kihara, 2014; Chapple et al., 2015; Hernández et al., 2015; Irving et al., in press). Our MoonProt Database can provide a test set for the further development of protein function prediction programs (Mani et al., 2015).

Additional knowledge about which proteins have multiple functions and roles in multiple biochemical pathways, multiprotein complexes or signaling pathways could also help in the prediction of protein function from comparative expression studies, protein-protein interaction analysis, gene knockout experiments, and other proteomics projects (Jeffery, 2014).

## Evolution of protein function and design of proteins with novel functions

How a second functional site evolved on a protein structure, as well as how regulation of protein expression, activity, and switching between functions evolved are interesting questions. In some cases, proteins were adopted for a second use without much change in physical features, but in other cases the evolution of new binding sites, new conformational changes, or other features were required. The paper by the DeLuna group in this Research Topic describes gene duplication in the evolution of moonlighting proteins (Espinosa-Cantu et al., in press).

Information about how moonlighting proteins evolved and information about their structures and functions can also be used to aid the design of proteins with new biochemical functions because they serve as examples of how to start with a stable protein fold as a scaffold and add a new functional site. They also provide examples of combining two functional sites in one polypeptide chain, which might be useful in the design of multifunctional protein therapeutics (or proteins used in manufacturing), to deliver one polypeptide drug that can perform multiple functions with those functional sites present at a selected stoichiometry.

## Summary

The examples above illustrate just some of the ways in which the continuing study of moonlighting proteins is important: developing novel therapeutics, identifying previously unknown cellular processes, elucidating the connections among biochemical pathways, understanding novel protein mechanisms, improving the prediction of protein functions, and providing information about the evolution of protein structure and function as well as examples for the design of new proteins. The references listed above and especially the collection of papers in this Research Topic are recommended for providing more in-depth examples and analysis of these topics.

### Conflict of interest statement

The author declares that the research was conducted in the absence of any commercial or financial relationships that could be construed as a potential conflict of interest.
